# AARS Online: A collaborative database on the structure, function, and evolution of the aminoacyl‐tRNA synthetases

**DOI:** 10.1002/iub.2911

**Published:** 2024-09-09

**Authors:** Jordan Douglas, Haissi Cui, John J. Perona, Oscar Vargas‐Rodriguez, Henna Tyynismaa, Claudia Alvarez Carreño, Jiqiang Ling, Lluís Ribas de Pouplana, Xiang‐Lei Yang, Michael Ibba, Hubert Becker, Frédéric Fischer, Marie Sissler, Charles W. Carter, Peter R. Wills

**Affiliations:** ^1^ Department of Physics University of Auckland New Zealand; ^2^ Centre for Computational Evolution University of Auckland New Zealand; ^3^ Department of Chemistry University of Toronto Canada; ^4^ Department of Chemistry Portland State University Portland Oregon USA; ^5^ Department of Molecular Biology and Biophysics University of Connecticut Storrs Connecticut USA; ^6^ Stem Cells and Metabolism Research Program, Faculty of Medicine University of Helsinki Finland; ^7^ Institute of Structural and Molecular Biology University College of London UK; ^8^ Department of Cell Biology and Molecular Genetics University of Maryland College Park Maryland USA; ^9^ Institute for Research in Biomedicine The Barcelona Institute of Science and Technology Barcelona Catalonia Spain; ^10^ Catalan Institution for Research and Advanced Studies Barcelona Catalonia Spain; ^11^ Department of Molecular Medicine The Scripps Research Institute La Jolla California USA; ^12^ Biological Sciences Chapman University Orange California USA; ^13^ Génétique Moléculaire, Génomique Microbiologique University of Strasbourg France; ^14^ Department of Biochemistry and Biophysics University of North Carolina at Chapel Hill Chapel Hill North Carolina USA

**Keywords:** aminoacyl‐tRNA synthetases, database, protein structure

## Abstract

The aminoacyl‐tRNA synthetases (aaRS) are a large group of enzymes that implement the genetic code in all known biological systems. They attach amino acids to their cognate tRNAs, moonlight in various translational and non‐translational activities beyond aminoacylation, and are linked to many genetic disorders. The aaRS have a subtle ontology characterized by structural and functional idiosyncrasies that vary from organism to organism, and protein to protein. Across the tree of life, the 22 coded amino acids are handled by 16 evolutionary families of Class I aaRS and 21 families of Class II aaRS. We introduce AARS Online, an interactive Wikipedia‐like tool curated by an international consortium of field experts. This platform systematizes existing knowledge about the aaRS by showcasing a taxonomically diverse selection of aaRS sequences and structures. Through its graphical user interface, AARS Online facilitates a seamless exploration between protein sequence and structure, providing a friendly introduction to the material for non‐experts and a useful resource for experts. Curated multiple sequence alignments can be extracted for downstream analyses. Accessible at www.aars.online, AARS Online is a free resource to delve into the world of the aaRS.

## 1 INTRODUCTION

The aminoacyl‐tRNA synthetases (aaRS) are a diverse group of enzymes that attach amino acids to their cognate tRNAs.^1^ These enzymes implement the genetic code in all known organisms, in all domains of life—the bacteria, archaea, and eukarya—as well as mitochondria and chloroplasts,^2^ and are also expressed by certain viruses.^3^ Due to their fundamental roles in building and managing living cells, aaRS mutants are linked to many diseases in higher organisms,^4^ where they carry out a wide range of additional functions that are not directly related to protein synthesis.^5^

Aminoacylation is a two‐step reaction powered by adenosine triphosphate (ATP).^6^ Adenosine monophosphate (AMP) and pyrophosphate (PP_*i*_) are released as byproducts of the reaction:

$$\begin{array}{l} \text{Amino acid activation}:\text{amino acid}+\mathrm{ATP}\\ \;\to \text{aminoacyl}-\mathrm{AMP}+{\mathrm{PP}}_{i} \end{array}$$

$$\begin{array}{l} \text{tRNA charging}:\text{aminoacyl}-\mathrm{AMP}+\text{tRNA}\\ \;\to \text{aminoacyl}-\text{tRNA}+\mathrm{AMP} \end{array}$$

These two reactions are carried out by the catalytic domain, which exists in two distinct evolutionary forms: Class I and Class II. The Class I catalytic domain is a Rossmann fold, with four parallel β‐strands, and Class II an anti‐parallel β‐sheet with six strands. Nine of the 22 coded amino acids are supplied to the ribosomal machinery from tRNAs (directly or indirectly) charged exclusively by Class I enzymes, 11 by Class II, and the remaining two amino acids, lysine^7^ and cysteine,^8^ can be rendered variously from the products of either Class I or II analogs. The second reaction, tRNA charging, is facilitated through recognition of the cognate tRNA by numerous structural elements of the catalytic domain,^9^ and one or more additional domains that bind to the anticodon stem,^10^ the D‐loop,^11^ the variable arm,^12^ or other parts of the tRNA molecule. Mis‐activated and mis‐charged amino acids can be expelled from the reaction pathway through editing activity, which operates at the pre‐transfer and post‐transfer level, respectively.^1^ Editing is necessary when varying amino acid types cannot be accurately distinguished by the active sites of the enzymes.^6^ Some aaRS possess an additional domain that catalyzes post‐transfer editing.^13^, ^14^

In most cases, each aaRS supplies just a single type of amino acid onto the growing peptide, and that amino acid is specified in the naming of the enzyme, for example alanyl‐tRNA synthetase (AlaRS) attaches alanine to tRNA^*Ala*^. However, certain aaRS can supply an additional amino acid or provide a building block, which is then elaborated into the amino acid corresponding to the tRNA. First, the metazoan glutamyl‐prolyl‐tRNA synthetase (EPRS) is a fusion of the catalytic domains from GluRS and ProRS connected by a linker and hence supplies both amino acids to their respective tRNA. Second, the original amino acid can be changed after its attachment to tRNA before reaching the ribosome. This is the case for the non‐discriminating aspartyl‐ and glutamyl‐tRNA synthetases (AsxRS and GlxRS), which attach Asp to tRNA^*Asn*^ and Glu to tRNA^*Gln*^, respectively.^15^, ^16^ The rare GluGlnRS attaches Glu to tRNA^*Gln*^ in a discriminating manner, representing an ancestral midpoint between the discriminating and non‐discriminating modes.^17^, ^18^ Likewise, O‐phosphoseryl‐tRNA synthetase (SepRS) supplies cysteine for organisms that lack CysRS,^8^ and SerRS charges serine to both tRNA^*Ser*^ and tRNA^*Sec*^.^19^ Selenocysteine (Sec) is the only amino acid whose tRNA is exclusively charged in this manner.

All human aaRS are encoded by nuclear genes, and all of them have been linked to genetic diseases.^4^, ^20^, ^21^, ^22^, ^23^, ^24^ Nineteen aaRS enzymes remain in the cytosol (EPRS doubles up) and 19 are relocated to mitochondria (no mitochondrial GlnRS).^20^ The cytosolic and mitochondrial aaRS are encoded by separate genes, with the exception of GlyRS and LysRS, which are shared by both compartments through dual targeting signals.^25^ Each protein is encoded by a single gene with the exception of the cytosolic PheRS protein, whose α and β subunits are expressed by two genes. Therefore humans have a total of 37 aaRS genes and 36 proteins (splice variants excluded). Genes that produce cytosolic and mitochondrial aaRS are conventionally named XARS1 and XARS2 respectively, for example, CARS1 and CARS2 encode cytosolic and mitochondrial cysteinyl‐tRNA synthetase, and the latter often resemble bacterial aaRS, due to the endosymbiotic origin of mitochondria.^26^ The dominant cytosolic aaRS mutations typically affect the peripheral nervous system (e.g., Charcot–Marie–Tooth disease^27^) while the recessive mutations in cytosolic aaRS tend to affect a large range of organs and are often accompanied with developmental delays (e.g., microcephaly^28^).^29^ Mitochondrial aaRS mutations typically affect organs of high metabolic demand, such as the brain and heart, as well as other tissues (e.g., leukodystrophy^30^).^22^, ^31^, ^32^ In some cases, mutations in the same aaRS result in strikingly different disease phenotypes, but in most cases the causal mutations of a disorder can occur at many positions in the respective aaRS, even across different domains.^21^

**FIGURE 1:**
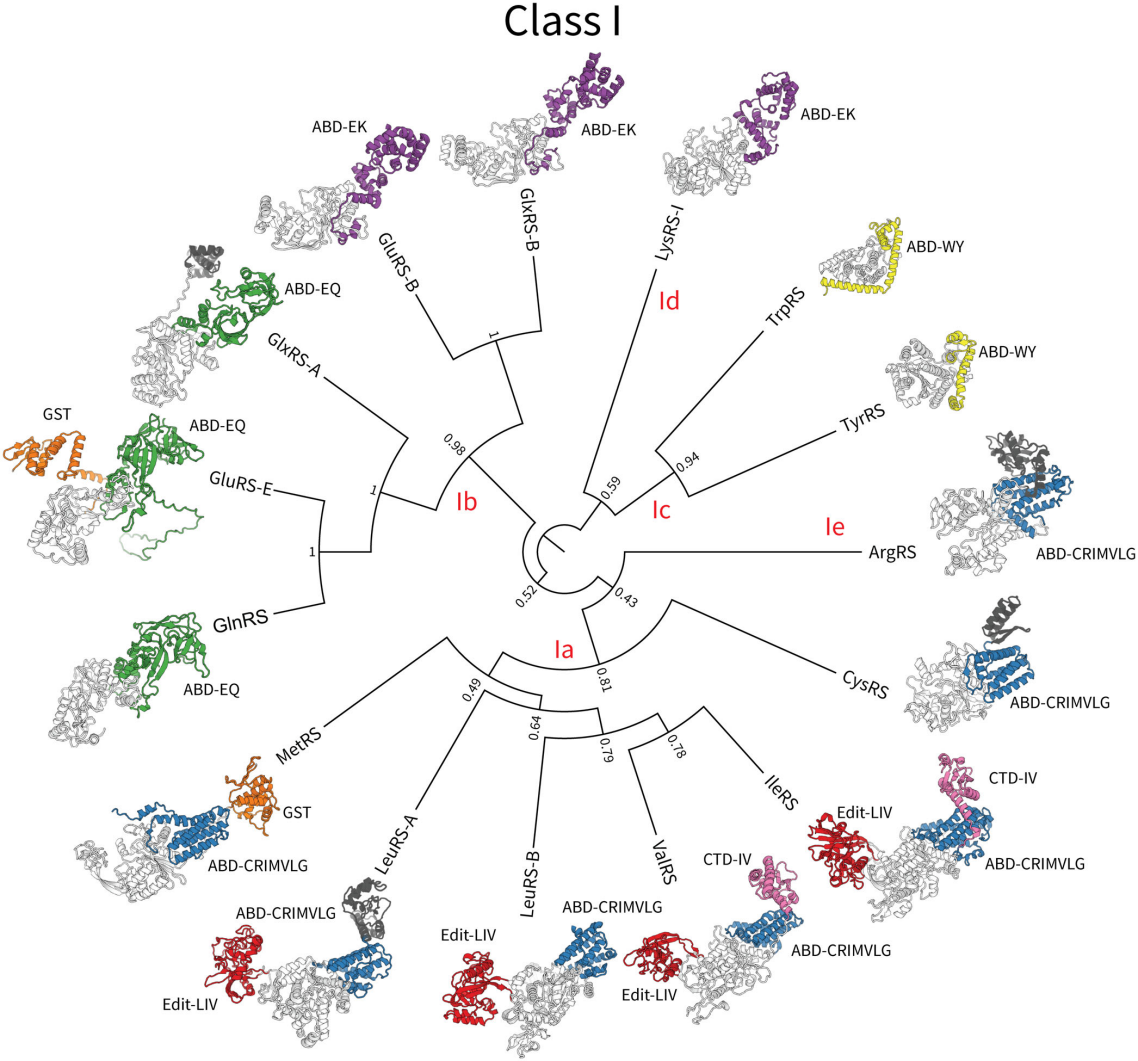
An inferred phylogeny of the Class I catalytic domain.^9^ Protein structures are colored by domain Superfamily—catalytic domain: light gray, ABD‐EK: purple, ABD‐EQ: green, ABD‐CRIMVLG: blue, ABD‐WY: yellow, Edit‐LIV: red, GST: orange, all other domains: dark gray. Branch lengths are proportional to amino acid substitutions per site, and internal nodes are labeled by clade posterior support. Please refer to Table 2 for domain Superfamilies.

**FIGURE 2:**
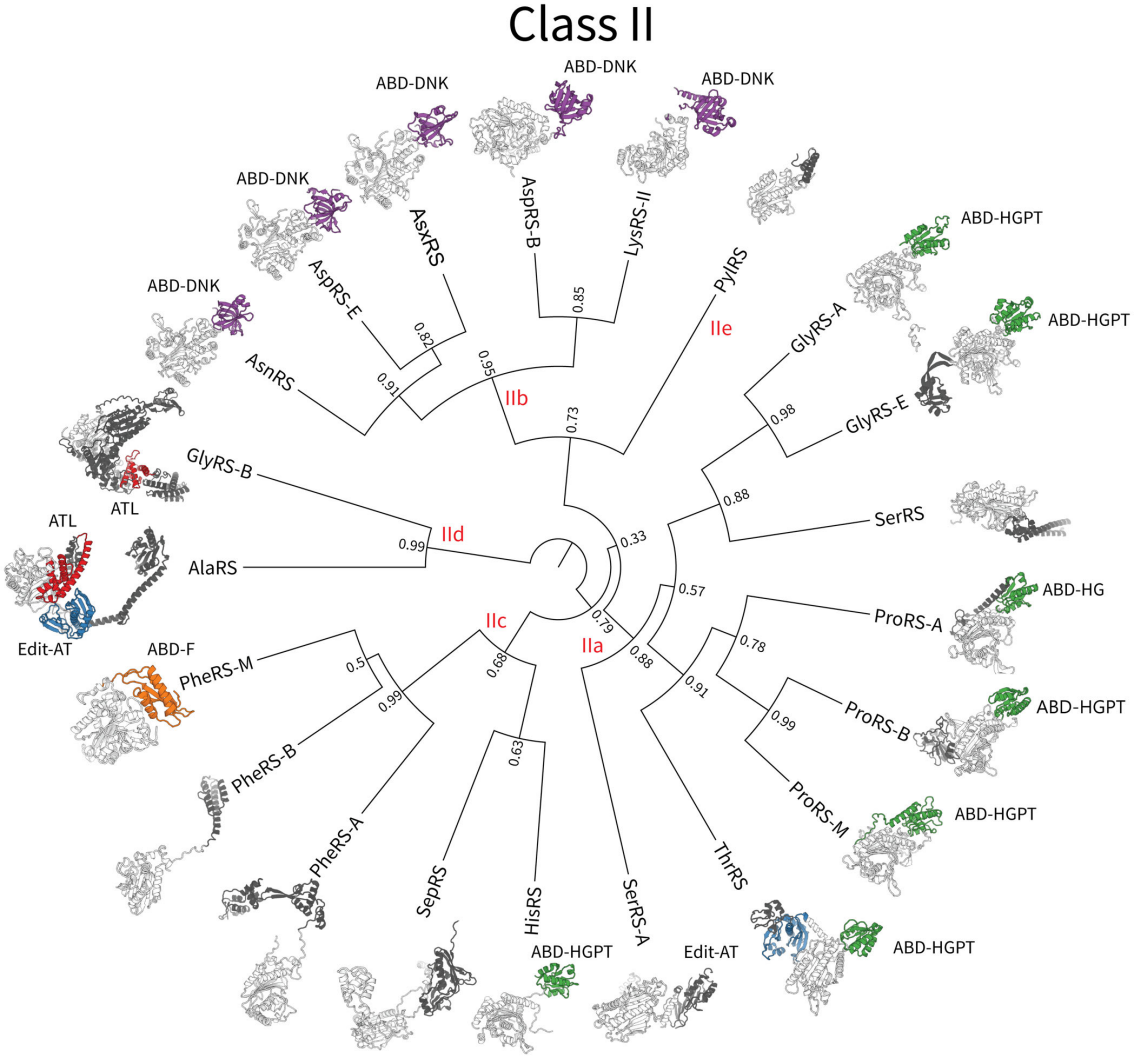
An inferred phylogeny of the Class II catalytic domain.^9^ Protein structures are colored by domain Superfamily—catalytic domain: light gray, ABD‐DNK: purple, ABD‐HGPT: green, ABD‐F: orange, Edit‐AT: blue, ATL: red, all other domains: dark gray. Branch lengths are proportional to amino acid substitutions per site, and internal nodes are labeled by clade posterior support. ABD‐DNK also occurs in some of the Class I aaRS in the form of EMAP. Note that the β chains of PheRS were omitted from this phylogeny, as their catalytic domain paralog has diverged from the rest of the Superfamily, and the β chains of GluRS‐B were omitted as they do not possess a paralog of the catalytic domain at all (the GlyRS‐B structure displayed is a fusion of the two chains). Please refer to Table 2 for domain Superfamilies.

Protein domains in general display a tendency to rearrange, duplicate, and exchange as functional modules on evolutionary timescales,^33^, ^34^, ^35^ and the aaRS are no exception to this phenomenon. Several aaRS have distinct isoforms that arose from gene fusion or fission events, such as the metazoan EPRS^36^ and the parasitic TyrRS,^37^ which each have two catalytic domains, and the bacterial GlyRS, which usually consists of α and β subunits α_2_β_2_, but occurs as an (αβ)_2_ fusion in some bacteria and chloroplasts.^38^, ^39^ Fused and fissed isoforms also exist for AlaRS,^40^ PheRS,^41^ and PylRS.^42^ These relatively recent gene rearrangement events shed light on deeper phylogenetic incongruences across the respective Class. Namely, LysRS‐I and GluRS have paralogous anticodon binding domains, but their catalytic domains are more divergent (Figure 1). And the anticodon binding domain of HisRS is related to ProRS and ThrRS, while its catalytic domain is more similar to PheRS (Figure 2). These “domain hopping” events have led to conflicting phylogenetic interpretations of LysRS‐I and HisRS in the past.^9^, ^43^, ^44^, ^45^, ^46^ Similarly, AlaX proteins remove mischarged amino acids from tRNA, and also appear as a C‐terminal domain of AlaRS and an N‐terminal domain of ThrRS,^47^, ^48^, ^49^ while various eukaryotic extensions, such as the GST‐like and WHEP domains, also occur elsewhere in the proteome.^50^

Accordingly, each aaRS is herein classified at three taxonomic levels: Class, Subclass, and Family, based on its catalytic domain (Table 1), which is the only domain common to the aaRS.^51^ First, there are two catalytic domain *Classes*: Class I and Class II. All members of a class share common ancestry and are therefore protein Superfamilies under the CATH and SCOP frameworks.^52^, ^53^ Second, aaRS are further divided into *Subclasses*, based on their sequence and structural similarities. However, there is no general consensus on subclass assignment.^1^, ^9^, ^43^, ^44^, ^45^, ^46^, ^54^ These discrepancies arise from a combination of faded phylogenetic signals (these proteins are over three billion years old), the use of varying phylogenetic methodologies and datasets, and also depend on whether the classification was based on the full‐length protein or the catalytic domain only. In any case, members of a subclass tend to recognize amino acids that have similar properties, for instance the branched chain amino acids (Leu, Ile, and Val) are all supplied by the enzymes of Subclass *Ia*, and large aromatic side chains (Trp and Tyr) by Subclass *Ic*. Third, following the nomenclature by Douglas et al.,^9^ a *Family* is a set of catalytic domains that have the same aminoacylation activity, are monophyletic (or are monophyletic with a second Family contained within them), and cannot be further distinguished by an insertion or deletion of at least 50 amino acids. In the case of multiple Families sharing the same function, they are named after their predominant taxonomic group: ‘A' for archaeal‐like, ‘B' for bacterial‐like, ‘E' for eukaryote‐like, and ‘M' for mitochondrial‐like. As the aaRS are often dual targeted to mitochondria and chloroplasts,^2^ with information on chloroplastic aaRS being scarce, the ‘M' moniker does not discriminate between either compartment. As a tiebreaker, the ‘A' label takes precedence over ‘E', as eukaryotes can be regarded as specialized forms of archaea.^55^ For instance, the archaeal/eukaryotic‐like ProRS is denoted by ProRS‐A. As shown in Table 1, there are many instances of common aminoacylation functions that fall under multiple Families, such as glycyl‐tRNA synthetase, which exists as bacterial‐like, archaeal‐like, and eukaryote‐like forms (GlyRS‐B, GlyRS‐A, and GlyRS‐E), and the two forms of leucyl‐tRNA synthetase (LeuRS‐A and LeuRS‐B) whose editing domains are nested in distinct regions of the catalytic domain.^56^

**TABLE 1:** Summary of aaRS Families.

Class	Subclass	Family	Activates	Supplies	PDB	Human genes	AARS Online
*I*	*a*	CysRS	Cys	Cys	1LI5^80^	CARS1, CARS2	aars.online/class1/cys
		IleRS	Ile	Ile	7D5C^81^	IARS1, IARS2	aars.online/class1/ile
		LeuRS‐A	Leu	Leu	1WZ2^56^	LARS1	aars.online/class1/leu2
		LeuRS‐B	Leu	Leu	2V0C^82^	LARS2	aars.online/class1/leu1
		MetRS	Met	Met	1PFV^83^	MARS1, MARS2	aars.online/class1/met
		ValRS	Val	Val	1GAX^84^	VARS1, VARS2	aars.online/class1/val
	*b*	GlnRS	Gln	Gln	1QTQ^85^	QARS1	aars.online/class1/gln
		GluGlnRS	Glu	Gln	−		aars.online/class1/glu1
		GluRS‐B	Glu	Glu	1J09^86^		aars.online/class1/glu1
		GlxRS‐B	Glu	Gln, Glu	2CFO^87^	EARS2	aars.online/class1/glu1
		GluRS‐E	Glu	Glu	7WRU^88^	EPRS1	aars.online/class1/glu3
		GlxRS‐A	Glu	Gln, Glu	3AII^89^		aars.online/class1/glu2
	*c*	TrpRS	Trp	Trp	1I6M^90^	WARS1, WARS2	aars.online/class1/trp
		TyrRS	Tyr	Tyr	2J5B^91^	YARS1, YARS2	aars.online/class1/tyr
	*d*	LysRS‐I	Lys	Lys	1IRX^7^		aars.online/class1/lys
	*e*	ArgRS	Arg	Arg	1BS2^92^	RARS1, RARS2	aars.online/class1/arg
*II*	*a*	GlyRS‐A	Gly	Gly	1ATI^93^		aars.online/class2/gly1
		GlyRS‐E	Gly	Gly	2Q5H^94^	GARS1	aars.online/class2/gly3
		ProRS‐A	Pro	Pro	1NJ8^95^	EPRS1	aars.online/class2/pro1
		ProRS‐B	Pro	Pro	2J3M^13^		aars.online/class2/pro2
		ProRS‐M	Pro	Pro	−	PARS2	aars.online/class2/pro2
		SerRS	Ser	Sel, Ser	1WLE^96^	SARS1, SARS2	aars.online/class2/ser1
		SerRS‐A	Ser	Ser	2CJB^97^		aars.online/class2/ser2
		ThrRS	Thr	Thr	1NYR^98^	TARS1, TARS2	aars.online/class2/thr
	*b*	AsnRS	Asn	Asn	1X54^99^	NARS1, NARS2	aars.online/class2/asn
		AspRS‐B	Asp	Asp	1C0A^100^	DARS2	aars.online/class2/asp1
		AspRS‐E	Asp	Asp	1EOV^101^	DARS1	aars.online/class2/asp2
		AsxRS	Asp	Asn, Asp	1B8A^102^		aars.online/class2/asp2
		LysRS‐II	Lys	Lys	3E9H^103^	KARS1	aars.online/class2/lys
	*c*	PheRS‐Aα	Phe	Phe	3L4G^104^	FARSA	aars.online/class2/phe3
		PheRS‐Aβ	Phe	Phe	3L4G^104^	FARSB	aars.online/class2/phe4
		PheRS‐Bα	Phe	Phe	3PCO^105^		aars.online/class2/phe1
		PheRS‐Bβ	Phe	Phe	3PCO^105^		aars.online/class2/phe2
		PheRS‐M	Phe	Phe	3CMQ^106^	FARS2	aars.online/class2/phe5
		HisRS	His	His	1HTT^107^	HARS1, HARS2	aars.online/class2/his
		SepRS	Sep	Cys	2ODR^108^		aars.online/class2/sep
	*d*	AlaRS	Ala	Ala	3HXV^47^	AARS1, AARS2	aars.online/class2/ala
		GlyRS‐Bα	Gly	Gly	1J5W^93^		aars.online/class2/gly2
	*e*	PylRS	Pyl	Pyl	2Q7E^109^		aars.online/class2/pyl

*Note*: Where available, an example of a solved structure is specified by its Protein Data Bank (PDB) code. The tetrameric PheRS contains paralogs of the Class II catalytic domain in the α and β chains, however catalysis is confined to the α subunit.^41^ In contrast, the β subunit of GlyRS‐B does not have a paralog of the catalytic domain, and hence does not have a web page.

Remarkably, the evolutionary history of the aaRS traces back to primordial life before the last universal common ancestor.^57^ Under the nucleopeptide world hypothesis (see reviews^58^, ^59^), the genetic code originated in an environment governed by the RNA catalysis of peptide synthesis, and the peptide catalysis of RNA synthesis. The seemingly‐unrelated Class I and II aaRS plausibly originated as proteins that co‐docked tRNA molecules from opposing sides,^43^ and encoded by opposing strands of a bidirectional gene,^60^, ^61^ which would have served as a central integrating role between the RNA and peptide populations.^61^, ^62^ These small, primitive aaRS, known as “urzymes”^63^, ^64^, ^65^, ^66^, ^67^ would have been promiscuous enzymes that may have recognized small, primitive forms of tRNA, known as “minihelices.”^68^, ^69^, ^70^, ^71^ Nucleopeptide world is a direct challenge to the classical RNA world hypothesis (see reviews^59^, ^72^), which postulates that genetic coding originated in a world governed by self‐replicating RNA catalysts, including ribozymal aaRS^73^ that were later supplanted by the proteinaceous forms we know today. Many laboratories have successfully engineered ribozymes that carry out one of the two aaRS reactions, but not both.^74^, ^75^, ^76^

There are representative solved structures for the majority of known aaRS Families (Table 1). However, given the extensive resources needed to produce just one structure, those available are often sourced from model organisms, or are chosen for their medical/economic relevance or their ability to crystallize, and are thus far from a representative sample of the biosphere's full diversity. For instance, the three most common source organisms on the entire Protein Data Bank are all eukaryotic, all multicellular, and all vertebrates—humans, cattle, and chickens (May 2024). Likewise, a disproportionately large number of solved aaRS structures were sourced from *Escherichia coli*, *Saccharomyces cerevisiae*, and *Thermus thermophilus*. The advent of AlphaFold^77^ means that protein structures can be accurately predicted, more so when the protein has solved homologs, and therefore these taxonomic sampling biases can be addressed. While AlphaFold models are still less reliable than experimentally determined structures,^78^, ^79^ they can be useful for generating experimentally testable hypotheses when considered in conjunction with other lines of evidence.

Existing knowledge about the aaRS is vast, and has been assembled from a range of academic perspectives, including enzymology, structural biology, cell biology, biomedicine, and phylogenetics. Navigating the plethora of aaRS Families across these diverse academic fields can be challenging. Here we introduce AARS Online—a platform that systematizes aaRS knowledge across the tree of life, built by, and for the use of, the aaRS community. At its core, AARS Online is a user‐friendly Wikipedia‐like tool for interactively displaying aaRS structures, sequences, and evolutionary information side‐by‐side. The platform is open‐source and hosted on GitHub (https://github.com/aarsonline/aarsonline.github.io), promoting users to edit and contribute content, ensuring that material remains up‐to‐date. This resource can be accessed at https://www.aars.online.

## 2 MATERIALS AND METHODS

### 2.1 Sequence and structure alignment

We selected a digestible number of sequences and structures for each aaRS Family—between 5 and 25. These samples consist of solved and predicted protein structures, sourced from both model organisms and the rest of the biosphere. We used the following inclusion criteria for selecting sequences/structures. First, we included experimentally solved aaRS structures from the Protein Data Bank. Second, we extracted additional annotated aaRS sequences from GenBank (see sampling protocol below) and predicted their monomeric structures using AlphaFold v2.3.0.^77^ Most of these AlphaFold models were generated by the New Zealand eScience Institute cluster, and others by ColabFold.^110^ We ensured that the aaRS from the following model organisms were included (as either solved or AlphaFold structures): *Escherichia coli*, *Homo sapiens* (cytosolic and mitochondrial), and *Saccharomyces cerevisiae* (cytosolic only). We randomly sampled additional sequences/structures for inclusion in a taxonomically‐representative fashion, such that each aaRS Family had a minimum of four samples from four phyla, and where possible, up to eight bacterial phyla, four archaeal phyla, four eukaryotic phyla, and one viral phylum, plus two organellar (mitochondrial or chloroplast) samples from two distinct eukaryotic phyla.

Protein three dimensional structures were displayed using PV.* Secondary structures were defined using DSSP v3.0.0.^111^, ^112^ Pairwise structural alignments were generated by DeepAlign.^113^ Per‐Family multiple sequence alignments were generated by first aligning the structures with 3DCOMB,^114^ followed by a refinement algorithm that realigned contiguous regions of at least three sites lacking secondary structure, using ClustalW^115^ based on primary structure.^9^ As existing structural alignment tools were not always reliable at delineating homologous insertions, alignments were then adjusted manually. Realignment and adjustment were especially important for the Class I KMSKS motif, whose structure is highly flexible and yet its sequence is strongly conserved.

Most of the protein structures displayed in Figures 1 and 2 are experimental structures, whose PDB codes are specified in Table 1. However, in order to capture additional domains that were missing from experimental structures, we used AlphaFold structures for the following Families: CysRS (species: *Aciduliprofundum boonei*; GenBank: 8827372), MetRS (*Scheffersomyces stipitis*; 4836672), GlyRS‐B (*Chlamydia pneumoniae*; 45051002), GluRS‐E (*Cyathus striatus*; KAF9013924), and GlxRS‐A (*Sulfolobus islandicus*; 15298417). This enabled the following domains to be visualized: a small C‐terminal extension of CysRS, the GST domain of MetRS and GluRS‐E, the small N‐terminal extension of GlxRS‐A, and the beta‐chain domain of GlyRS‐B. Cognate tRNA secondary structures were extracted from tRNAViz.^116^

### 2.2 Phylogenetics

The catalytic domain phylogenies in Figures 1 and 2 are from Douglas et al.^9^ and were displayed using FigTree.† The remaining phylogenetic analyses presented on AARS Online were performed using BEAST v2.7.6.^117^ All phylogenies presented on AARS Online were inferred under the optimized relaxed clock (ORC v1.2.0),^118^ the OBAMA substitution model (OBAMA v1.1.1),^119^ and the Yule Skyline tree prior (BICEPS v1.1.1).^120^ Trees were summarized using the CCD0 tree.^121^ Markov chain Monte Carlo was run until the effective sample sizes of all parameters exceeded 200, diagnosed using Tracer v1.6.^122^ The cross‐Family catalytic domain phylogenies were constructed using the common elements of the catalytic domain of either Class. The OBAMA method selected the WAG substitution model^123^ for Class I (with 100% posterior support), and the LG model^124^ for Class II (also with 100% support).

### 2.3 Web content

AARS Online is hosted on GitHub and was coded entirely in JavaScript. All computation and database indexing are performed client‐side, which avoids congestion at a back‐end server. Curators are able to edit the web page introductions by editing markdown files on GitHub, which automatically deploys any changes made. Users are able to submit pull requests to make changes, which will be deployed pending acceptance by a curator. We anticipate pull requests to predominantly take on the following forms: (a) contribution to the introductory text at the top of each page, and (b) adjustment to the protein domain annotations. We will also consider requests to add novel sequences to existing pages (or new pages altogether) in a manner that does not impair the simplicity or reliability of the platform. A request to add a new sequence is more likely to be accepted if it contains a domain absent from the other displayed proteins, for instance.

## 3 RESULTS AND DISCUSSION

### 3.1 Sequence and structural data

In total, AARS Online includes 545 aaRS structures: 68 were extracted from the Protein Data Bank and 477 were generated using AlphaFold. This taxonomically diverse sample was sourced from 49 phyla of the tree of life: 228 structures are bacterial (from 17 bacterial phyla), 113 archaeal (8 phyla), 137 from the eukaryotic cytosol (17 phyla), 60 from the eukaryotic organelles (14 phyla), and 7 viral (single phylum). This viral phylum corresponds to the giant viruses, which have collected several aaRS in their abnormally large genome.^3^ Although taxonomy is dynamic, especially in the case of archaea, and many life forms remain undiscovered or unsequenced, much of the aaRS diversity across the tree of life has likely been captured by this sample.

The reliability of the AlphaFold models can be evaluated on AARS Online by displaying the per‐site pLDDT scores on the 3D protein structures. The pLDDT scores were generally quite large, providing some level of confidence in the structural models. All aaRS Families had a median score of at least 94% for the catalytic domain and at least 91% for the full‐length protein. The α chain of PheRS‐A was the lowest scoring aaRS Family, whose catalytic domains had a median score of 95%, but the full‐length proteins were slightly lower at 91% due to the flexible linker between the two domains. The generally high scores reflect the abundance of experimentally solved aaRS structures, which were used to train AlphaFold. Low scoring regions of the aaRS generally correspond to domain linkers, and flexible or disordered regions,^9^ such as the KMSKS motif of Class I^125^ and the flexible loop found on the surface of CysRS.^80^

### 3.2 Website layout

In AARS Online, there is one web page per Family (see Table 1), and one per aaRS domain Superfamily that is represented by more than one Family (see Table 2). The pairwise alignment web pages provide comparisons between Families. These web pages contain a pairwise multiple sequence/structure alignment between every pair of catalytic domain Families from the same Class. Unlike the Family and domain pages, the content on the pairwise pages was automatically generated, and they were not treated to any level of manual curation or documentation.

**TABLE 2:** aaRS domain Superfamilies.

Superfamily	Description	Structure	Members	AARS Online
Class I	Catalytic domain	Rossmann fold	ArgRS, CysRS, GluGlnRS, GluRS‐B, GluRS‐E, GlnRS, GlxRS‐A, GlxRS‐B, LysRS‐I, IleRS, LeuRS‐A, LeuRS‐B, MetRS, TyrRS, TypRS, ValRS	aars.online/d/cat1
Class II	Catalytic domain	β‐Sheet	AlaRS, AsnRS, AspRS‐B, AspRS‐E, AsxRS, GlyRS‐A, GlyRS‐B, GlyRS‐E, HisRS, LysRS‐II, PheRS‐A, PheRS‐B, PheRS‐M, ProRS‐A, ProRS‐B, ProRS‐M, PylRS, SepRS, SerRS, SerRS‐A, ThrRS	aars.online/d/cat2
ABD‐CRIMVLG	Anticodon binding	Helix bundle	ArgRS, CysRS, IleRS, LeuRS‐A, LeuRS‐B, MetRS, ValRS, GlyRS‐B	aars.online/d/crimvlg
ABD‐EK	Anticodon binding	α‐Helical	GluRS‐B, GlxRS‐B, LysRS‐I	aars.online/d/ek
ABD‐EQ	Anticodon binding	β‐Barrel	GlnRS, GluRS‐E, GlxRS‐A	aars.online/d/eq
ABD‐WY	Anticodon binding	Helical	TrpRS, TyrRS	aars.online/d/wy
ABD‐HGPT	Anticodon binding	β‐Sheet	HisRS, GlyRS‐A, GlyRS‐E, ProRS‐A, ProRS‐B, ProRS‐M, ThrRS	aars.online/d/hgpt
ABD‐DNK	Anticodon binding EMAP	OB‐fold	AsxRS, AspRS‐B, AspRS‐E, AsnRS, LysRS‐II, MetRS, PheRS‐B*β*, TyrRS	aars.online/d/dnk
ABD‐F	Anticodon binding	α/β	PheRS‐Bβ, PheRS‐M	aars.online/d/f
CTD‐IV	C‐terminal junction	α/β	IleRS, ValRS	aars.online/d/iv
ATL	Ancient tRNA latch	Helix bundle	AlaRS, GlyRS‐Bα or β	aars.online/d/atl
Edit‐LIV	Post‐transfer editing	Globular	IleRS, LeuRS‐A, LeuRS‐B, ValRS	aars.online/d/edit1a
Edit‐AT	Post‐transfer editing	Globular	AlaRS, ThrRS	aars.online/d/editat
GST	Glutathione S‐transferase	α/β	CysRS, GluRS‐E, MetRS, ValRS	aars.online/d/gst
WHEP	WHEP domain	Helix bundle	GluRS‐E, GlyRS‐E, HisRS, MetRS, ProRS‐A, TrpRS	aars.online/d/whep

*Note*: Shown are domains that occur in at least two of the Families presented in Table 1. Each row in this table is accompanied by a web page in AARS Online. The ATL domain occurs in the β chain of most α_2_β_2_ GlyRS‐B proteins, and in the fused (αβ)_2_ protein in some bacteria and chloroplasts.^133^

**FIGURE 3:**
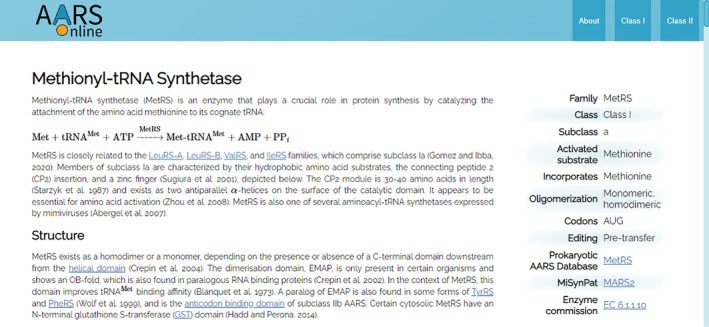
Screenshot of the introduction on the MetRS web page.

Each Family and domain web page begins with an introduction that provides a fully referenced mini‐review of the system (Figure 3). These introductions cover the following topics where applicable: structure and function, editing, non‐translational functions, clinical significance, and tRNA. The clinical significance sections discuss, where appropriate, the role that the cytosolic and mitochondrial aaRS genes play in human disease, and their role in antibiotic resistance. As AARS Online is hosted on GitHub, changes to the database and documentation are automatically tracked. To promote user engagement, each web page features a hyperlink to GitHub, where users are encouraged to initiate discussion or provide corrections and updates by submitting pull requests.

**FIGURE 4:**
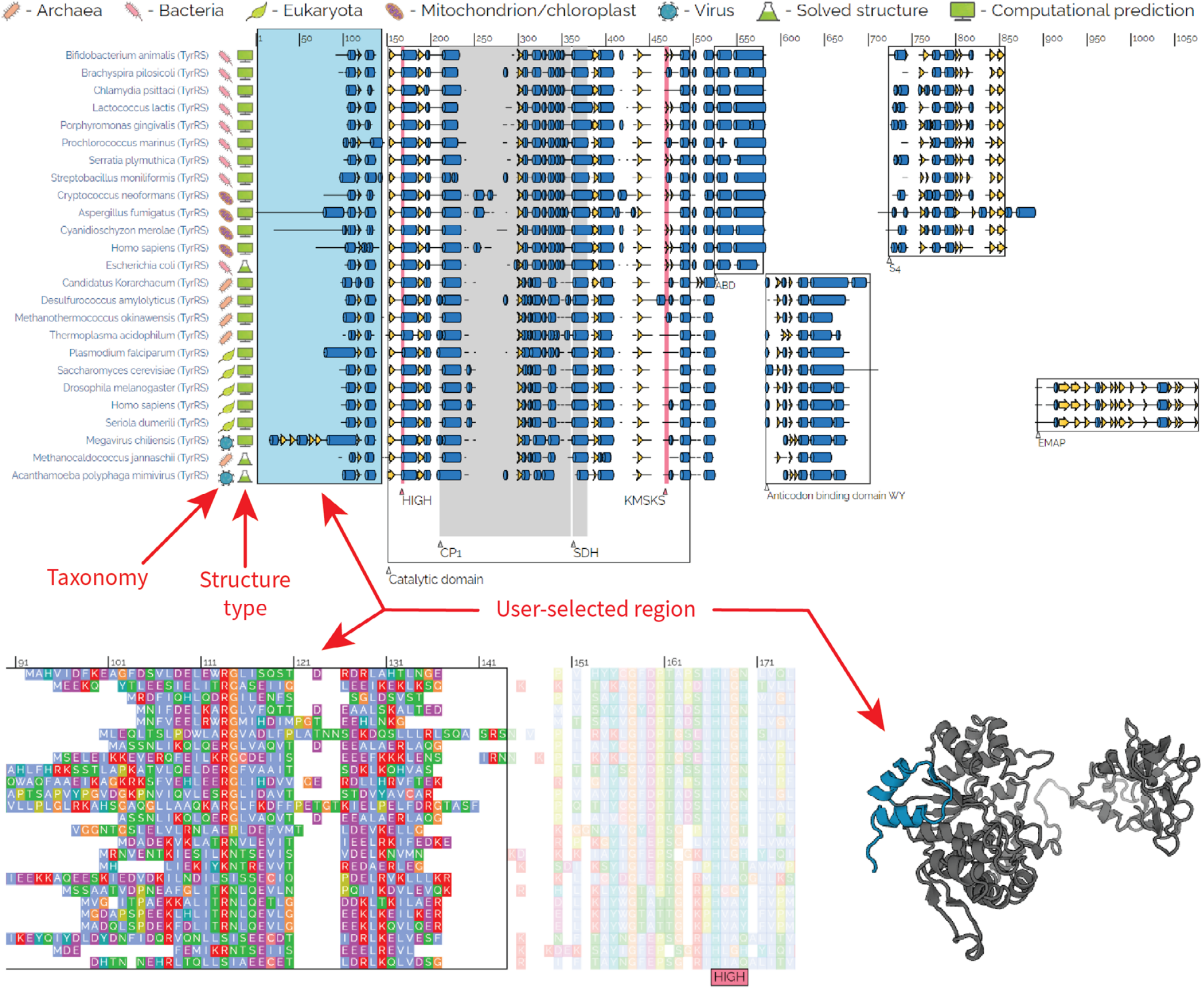
AARS Online display for a multiple sequence/structure alignment of TyrRS. Top: an alignment of domain architectures for TyrRS, HIGH, and KMSKS motifs annotated, as identified by Eriani et al.^127^ Notation—blue cylinders: helices; yellow arrows: β‐strands, black lines: loops/turns, white space: alignment gaps. Bottom left: the same alignment as above, but zoomed in to show the amino acid sequences. Bottom right: cartoon representation of an AlphaFold prediction for the *Homo sapiens* cytosolic TyrRS. The sequence and structure are readily navigated by dragging the cursor over this alignment: the user‐selected blue rectangle (top) corresponds to the highlighted regions on the primary structure and 3D protein cartoon (bottom).

Further down the page are annotated multiple sequence and structure alignments, a phylogenetic tree describing the relationships between proteins, and cognate tRNA secondary structures (with identity elements indicated, based on Giegé and Eriani^126^). The aaRS structures displayed are a combination of AlphaFold structures, subject to representative sampling from all domains of life (including organelles and viruses), and experimentally solved structures, subject to their availability. Sites in the multiple sequence alignment can be selected, and the corresponding areas on the protein structure(s) are also highlighted, allowing for a seamless exploration between sequence and structure (Figure 4). Primary and secondary structure alignments are displayed—the former is represented by the 20 canonical amino acid characters (plus gaps), and the latter by the eight DSSP secondary structure characters (plus gaps). These eight characters are: E—extended β‐strand; H—α‐helix; G—310 helix; I—p‐helix; B—b‐bridge; S—bend; T—H‐bonded turn; and N—other/unclassified.^111^, ^112^ These sequence and structural alignments can be downloaded and further interpreted with other software.

Most of the enzymes exhibited on AARS Online were assigned putative functions based on evolutionary analysis, without direct experimental validation, and therefore caution is advised during interpretation. We also advise a level of skepticism when interpreting the classification of eukaryotic aaRS into cytosolic and mitochondrial/chloroplastic compartments, or when eukaryotic splice sites have been predicted without support from mRNA sequencing data.

### 3.3 Related databases

Related databases and services include:

1. *2001*: *Aminoacyl‐tRNA Synthetase Data Bank (AARSDB)*.^128^ This resource provided annotated aaRS sequence data (at the time of writing, we could no longer access the AARSDB web‐server).
2. *2017*: *Mitochondrial Aminoacyl‐tRNA Synthetases & Pathologies (MiSynPat)*.^21^ MiSynPat documents the disease‐causing mutations linked to human mitochondrial aaRS, and much like AARS Online, facilitates ready navigation between sequence and structure. MiSynPat is available at misynpat.org.
3. *2017*: *Prokaryotic AARS Database*.^129^ This tool contains a series of aaRS sequence motifs to assist researchers in predicting aaRS functions based on sequence and is available at bioinf.bio.uth.gr/aars.
4. *2019*: *tRNAviz*.^116^ tRNAviz provides annotated tRNA structural annotations across the tree of life, allowing the user to identify conserved elements filtered by clade or tRNA isoform. tRNAviz is available at trna.ucsc.edu/tRNAviz/.

All structures on AARS Online are hyperlinked to their sources on GenBank (for AlphaFold structures) or the Protein Data Bank (for experimental structures). The codon table used during translation is specified, according to The Genetic Codes by NCBI,‡ which was compiled from earlier work.^130^, ^131^ Moreover, aaRS Families are hyperlinked to their corresponding entries in the Prokaryotic AARS Database^129^ and MiSynPat.^21^ Enzyme commission numbers are used to link the aaRS to the BRENDA enzyme database.^132^

## 4 OUTLOOK AND LIMITATIONS

AARS Online provides a friendly introduction to the aaRS and highlights the subtle idiosyncrasies that exist across the tree of life, and across the tree of aaRS. Enzymes are classified into Classes, Subclasses, and Families according to their catalytic domain(s), which is the only structural feature universal to the aaRS. While great care is taken to ensure its accuracy, a healthy level of skepticism is advised when navigating the platform, as many entries in the database are based on bioinformatic predictions: including aaRS functional assignments, gene boundaries, compartmentalization (cytosol or mitochondria/chloroplast), and protein structures (AlphaFold). While experimentally solved structures are generally not faced with the same limitations, they are much fewer in number, often harbor missing residues or truncated domains, and tend to be sourced from a comparatively narrow spectrum of the biosphere. Notwithstanding these caveats, we believe AARS Online to be a useful resource for experts and non‐experts alike. The recent release of AlphaFold3,^134^ and its ability to predict protein–RNA interactions, presents exciting future prospects for aaRS modeling. With the help of community input, we hope this platform will continue to evolve so that it always reflects the latest research around the aaRS.

## CONFLICT OF INTEREST STATEMENT

The authors have no conflicts of interest to declare.

## Footnotes

* Marco Biasini. (2015). pv: v1.8.1. Zenodo. 10.5281/zenodo.20980, available online at https://biasmv.github.io/pv/.

† FigTree available at http://tree.bio.ed.ac.uk/software/figtree/.

‡ Codon tables are summarized at https://www.ncbi.nlm.nih.gov/Taxonomy/Utils/wprintgc.cgi.

## References

[1] Gomez MAR , Ibba M . Aminoacyl‐tRNA synthetases. RNA. 2020;26(8):910–936. 32303649 10.1261/rna.071720.119 PMC7373986

[2] Duchêne A‐M , Giritch A , Hoffmann B , Cognat V , Lancelin D , Peeters NM , et al. Dual targeting is the rule for organellar aminoacyl‐tRNA synthetases in *Arabidopsis thaliana* . Proc Natl Acad Sci. 2005;102(45):16484–16489. 16251277 10.1073/pnas.0504682102 PMC1283425

[3] Arslan D , Legendre M , Seltzer V , Abergel C , Claverie J‐M . Distant Mimivirus relative with a larger genome highlights the fundamental features of Megaviridae. Proc Natl Acad Sci. 2011;108(42):17486–17491. 21987820 10.1073/pnas.1110889108 PMC3198346

[4] Antonellis A , Green ED . The role of aminoacyl‐tRNA synthetases in genetic diseases. Annu Rev Genomics Hum Genet. 2008;9:87–107. 18767960 10.1146/annurev.genom.9.081307.164204

[5] Guo M , Schimmel P . Essential nontranslational functions of tRNA synthetases. Nat Chem Biol. 2013;9(3):145–153. 23416400 10.1038/nchembio.1158 PMC3773598

[6] Perona JJ , Gruic‐Sovulj I . Synthetic and editing mechanisms of aminoacyl‐tRNA synthetases. Aminoacyl‐tRNA synthetases in biology and medicine. Dordrecht: Springer Netherlands; 2014. p. 1–41. 10.1007/128_2013_456 23852030

[7] Terada T , Nureki O , Ishitani R , Ambrogelly A , Ibba M , Söll D , et al. Functional convergence of two lysyl‐tRNA synthetases with unrelated topologies. Nat Struct Biol. 2002;9(4):257–262. 11887185 10.1038/nsb777

[8] Sauerwald A , Zhu W , Major TA , Roy H́ , Palioura S , Jahn D , et al. RNA‐dependent cysteine biosynthesis in archaea. Science. 2005;307(5717):1969–1972. 15790858 10.1126/science.1108329

[9] Douglas J , Bouckaert R , Carter CW Jr , Wills PR . Enzymic recognition of amino acids drove the evolution of primordial genetic codes. Nucleic Acids Res. 2024;52(2):558–571. 38048305 10.1093/nar/gkad1160 PMC10810186

[10] Charron C , Roy H , Blaise M , Giegé R , Kern D . Non‐discriminating and discriminating aspartyl‐tRNA synthetases differ in the anticodon‐binding domain. EMBO J. 2003;22(7):1632–1643. 12660169 10.1093/emboj/cdg148 PMC152893

[11] Konno M , Sumida T , Uchikawa E , Mori Y , Yanagisawa T , Sekine SI , et al. Modeling of tRNA‐assisted mechanism of arg activation based on a structure of arg‐tRNA synthetase, tRNA, and an atp analog (anp). FEBS J. 2009;276(17):4763–4779. 19656186 10.1111/j.1742-4658.2009.07178.x

[12] Dock‐Bregon A‐C , Garcia A , Giegé R , Moras D . The contacts of yeast tRNASer with seryl‐tRNA synthetase studied by footprinting experiments. Eur J Biochem. 1990;188(2):283–290. 2180700 10.1111/j.1432-1033.1990.tb15401.x

[13] Crepin T , Yaremchuk A , Tukalo M , Cusack S . Structures of two bacterial prolyl‐tRNA synthetases with and without a cis‐editing domain. Structure. 2006;14(10):1511–1525. 17027500 10.1016/j.str.2006.08.007

[14] Zhou X‐L , Zhu B , Wang E‐D . The cp2 domain of leucyl‐tRNA synthetase is crucial for amino acid activation and post‐transfer editing. J Biol Chem. 2008;283(52):36608–36616. 18955487 10.1074/jbc.M806745200 PMC2662312

[15] Lapointe J , Duplain L , Proulx M . A single glutamyl‐tRNA synthetase aminoacylates tRNAGlu and tRNAGln in *Bacillus subtilis* and efficiently misacylates *Escherichia coli* tRNAGln1 in vitro. J Bacteriol. 1986;165(1):88–93. 3079749 10.1128/jb.165.1.88-93.1986 PMC214374

[16] Raczniak G , Becker HD , Min B , Soll D . A single amidotransferase forms asparaginyl‐tRNA and glutaminyl‐tRNA in *Chlamydia trachomatis* . J Biol Chem. 2001;276(49):45862–45867. 11585842 10.1074/jbc.M109494200

[17] Skouloubris S , Ribas de Pouplana L , De Reuse H , Hendrickson TL . A noncognate aminoacyl‐tRNA synthetase that may resolve a missing link in protein evolution. Proc Natl Acad Sci. 2003;100(20):11297–11302. 13679580 10.1073/pnas.1932482100 PMC208751

[18] Salazar JC , Ahel I , Orellana O , Tumbula‐Hansen D , Krieger R , Daniels L , et al. Coevolution of an aminoacyl‐tRNA synthetase with its tRNA substrates. Proc Natl Acad Sci. 2003;100(24):13863–13868. 14615592 10.1073/pnas.1936123100 PMC283512

[19] Lee BJ , Worland PJ , Davis JN , Stadtman TC , Hatfield DL . Identification of a selenocysteyl‐tRNASer in mammalian cells that recognizes the nonsense codon, UGA. J Biol Chem. 1989;264(17):9724–9727. 2498338

[20] Fine AS , Nemeth CL , Kaufman ML , Fatemi A . Mitochondrial aminoacyl‐tRNA synthetase disorders: an emerging group of developmental disorders of myelination. J Neurodev Disord. 2019;11:1–15. 31839000 10.1186/s11689-019-9292-y PMC6913031

[21] Moulinier L , Ripp R , Castillo G , Poch O , Sissler M . MiSynPat: an integrated knowledge base linking clinical, genetic, and structural data for disease‐causing mutations in human mitochondrial aminoacyl‐tRNA synthetases. Hum Mutat. 2017;38(10):1316–1324. 28608363 10.1002/humu.23277 PMC5638098

[22] Turvey AK , Horvath GA , Cavalcanti AR . Aminoacyl‐tRNA synthetases in human health and disease. Front Physiol. 2022;13:1029218. 36330207 10.3389/fphys.2022.1029218 PMC9623071

[23] Konovalova S , Tyynismaa H . Mitochondrial aminoacyl‐tRNA synthetases in human disease. Mol Genet Metab. 2013;108(4):206–211. 23433712 10.1016/j.ymgme.2013.01.010

[24] Tyynismaa H . Disease models of mitochondrial aminoacyl‐tRNA synthetase defects. J Inherit Metab Dis. 2023;46(5):817–823. 37410890 10.1002/jimd.12652

[25] Gile GH , Moog D , Slamovits CH , Maier U‐G , Archibald JM . Dual organellar targeting of aminoacyl‐tRNA synthetases in diatoms and cryptophytes. Genome Biol Evol. 2015;7(6):1728–1742. 25994931 10.1093/gbe/evv095 PMC4494062

[26] Brindefalk B , Viklund J , Larsson D , Thollesson M , Andersson SG . Origin and evolution of the mitochondrial aminoacyl‐tRNA synthetases. Mol Biol Evol. 2007;24(3):743–756. 17182897 10.1093/molbev/msl202

[27] Wei N , Zhang Q , Yang X‐L . Neurodegenerative charcot–marie–tooth disease as a case study to decipher novel functions of aminoacyl‐tRNA synthetases. J Biol Chem. 2019;294(14):5321–5339. 30643024 10.1074/jbc.REV118.002955 PMC6462521

[28] Kuo ME , Theil AF , Kievit A , Malicdan MC , Introne WJ , Christian T , et al. Cysteinyl‐tRNA synthetase mutations cause a multi‐system, recessive disease that includes microcephaly, developmental delay, and brittle hair and nails. Am J Hum Genet. 2019;104(3):520–529. 30824121 10.1016/j.ajhg.2019.01.006 PMC6407526

[29] Jiang L , Jones J , Yang X‐L . Human diseases linked to cytoplasmic aminoacyl‐tRNA synthetases. Enzymes. 2020;48:277–319. 33837707 10.1016/bs.enz.2020.06.009

[30] Steenweg ME , Ghezzi D , Haack T , Abbink TEM , Martinelli D , van Berkel CGM , et al. Leukoencephalopathy with thalamus and brainstem involvement and high lactate ‘ltbl' caused by ears2 mutations. Brain. 2012;135(5):1387–1394. 22492562 10.1093/brain/aws070

[31] Yoon I , Kim U , Choi J , Kim S . Disease association and therapeutic routes of aminoacyl‐tRNA synthetases. Trends Mol Med. 2024;30:89–105. 37949787 10.1016/j.molmed.2023.10.006

[32] Sissler M , González‐Serrano LE , Westhof E . Recent advances in mitochondrial aminoacyl‐tRNA synthetases and disease. Trends Mol Med. 2017;23(8):693–708. 28716624 10.1016/j.molmed.2017.06.002

[33] Moore AD , Björklund ÅK , Ekman D , Bornberg‐Bauer E , Elofsson A . Arrangements in the modular evolution of proteins. Trends Biochem Sci. 2008;33(9):444–451. 18656364 10.1016/j.tibs.2008.05.008

[34] Bornberg‐Bauer E , Albà MM . Dynamics and adaptive benefits of modular protein evolution. Curr Opin Struct Biol. 2013;23(3):459–466. 23562500 10.1016/j.sbi.2013.02.012

[35] Dohmen E , Klasberg S , Bornberg‐Bauer E , Perrey S , Kemena C . The modular nature of protein evolution: domain rearrangement rates across eukaryotic life. BMC Evol Biol. 2020;20(1):1–13. 32059645 10.1186/s12862-020-1591-0 PMC7023805

[36] Eswarappa SM , Potdar AA , Sahoo S , Sankar S , Fox PL . Metabolic origin of the fused aminoacyl‐tRNA synthetase, glutamyl‐prolyl‐tRNA synthetase. J Biol Chem. 2018;293(49):19148–19156. 30309984 10.1074/jbc.RA118.004276 PMC6295713

[37] Larson ET , Kim JE , Castaneda LJ , Napuli AJ , Zhang Z , Fan E , et al. The double‐length tyrosyl‐tRNA synthetase from the eukaryote leishmania major forms an intrinsically asymmetric pseudo‐dimer. J Mol Biol. 2011;409(2):159–176. 21420975 10.1016/j.jmb.2011.03.026 PMC3095712

[38] Wagar EA , Giese MJ , Yasin B , Pang M . The glycyl‐tRNA synthetase of chlamydia trachomatis. J Bacteriol. 1995;177(17):5179–5185. 7665503 10.1128/jb.177.17.5179-5185.1995 PMC177304

[39] Uwer U , Willmitzer L , Altmann T . Inactivation of a glycyl‐tRNA synthetase leads to an arrest in plant embryo development. Plant Cell. 1998;10(8):1277–1294. 9707529 10.1105/tpc.10.8.1277 PMC144065

[40] Arutaki M , Kurihara R , Matsuoka T , Inami A , Tokunaga K , Ohno T , et al. G: U‐independent rna minihelix aminoacylation by nanoarchaeum equitans alanyl‐tRNA synthetase: an insight into the evolution of aminoacyl‐tRNA synthetases. J Mol Evol. 2020;88:501–509. 32382786 10.1007/s00239-020-09945-1 PMC11392972

[41] Klipcan L , Finarov I , Moor N , Safro MG . Structural aspects of phenylalanylation and quality control in three major forms of phenylalanyl‐tRNA synthetase. J Amino Acids. 2010;2010:1–7. 10.4061/2010/983503 PMC3275996 22331999

[42] Suzuki T , Miller C , Guo LT , Ho JML , Bryson DI , Wang YS , et al. Crystal structures reveal an elusive functional domain of pyrrolysyl‐tRNA synthetase. Nat Chem Biol. 2017;13(12):1261–1266. 29035363 10.1038/nchembio.2497 PMC5698177

[43] Ribas de Pouplana L , Schimmel P . Aminoacyl‐tRNA synthetases: potential markers of genetic code development. Trends Biochem Sci. 2001;26(10):591–596. 11590011 10.1016/s0968-0004(01)01932-6

[44] O'Donoghue P , Luthey‐Schulten Z . On the evolution of structure in aminoacyl‐tRNA synthetases. Microbiol Mol Biol Rev. 2003;67(4):550–573. 14665676 10.1128/MMBR.67.4.550-573.2003 PMC309052

[45] Perona JJ , Hadd A . Structural diversity and protein engineering of the aminoacyl‐tRNA synthetases. Biochemistry. 2012;51(44):8705–8729. 23075299 10.1021/bi301180x

[46] Carter CW , Popinga A , Bouckaert R , Wills PR . Multidimensional phylogenetic metrics identify Class I aminoacyl‐tRNA synthetase evolutionary mosaicity and inter‐modular coupling. Int J Mol Sci. 2022;23(3):1520. 35163448 10.3390/ijms23031520 PMC8835825

[47] Guo M , Chong YE , Shapiro R , Beebe K , Yang X‐L , Schimmel P . Paradox of mistranslation of serine for alanine caused by AlaRS recognition dilemma. Nature. 2009;462(7274):808–812. 20010690 10.1038/nature08612 PMC2799227

[48] Beebe K , Mock M , Merriman E , Schimmel P . Distinct domains of tRNA synthetase recognize the same base pair. Nature. 2008;451(7174):90–93. 18172502 10.1038/nature06454

[49] Nagy ABK , Bakhtina M , Musier‐Forsyth K . Trans‐editing by aminoacyl‐tRNA synthetase‐like editing domains. Enzymes. 2020;48:69–115. 33837712 10.1016/bs.enz.2020.07.002

[50] Guo M , Yang X‐L . Architecture and metamorphosis. Aminoacyl‐tRNA synthetases in biology and medicine. Dordrecht: Springer Netherlands; 2014. p. 89–118.

[51] Wolf YI , Aravind L , Grishin NV , Koonin EV . Evolution of aminoacyl‐tRNA synthetases—analysis of unique domain architectures and phylogenetic trees reveals a complex history of horizontal gene transfer events. Genome Res. 1999;9(8):689–710. 10447505

[52] Orengo CA , Michie AD , Jones S , Jones DT , Swindells MB , Thornton JM . CATH—a hierarchic classification of protein domain structures. Structure. 1997;5(8):1093–1109. 9309224 10.1016/s0969-2126(97)00260-8

[53] Andreeva A , Kulesha E , Gough J , Murzin AG . The scop database in 2020: expanded classification of representative family and superfamily domains of known protein structures. Nucleic Acids Res. 2020;48(D1):D376–D382. 31724711 10.1093/nar/gkz1064 PMC7139981

[54] Valencia‐Sánchez MI , Rodríguez‐Hernández A , Ferreira R , Santamaría‐Suárez HA , Arciniega M , Dock‐Bregeon AC , et al. Structural insights into the polyphyletic origins of glycyl tRNA synthetases. J Biol Chem. 2016;291(28):14430–14446. 27226617 10.1074/jbc.M116.730382 PMC4938167

[55] Zaremba‐Niedzwiedzka K , Caceres EF , Saw JH , Bäckström D , Juzokaite L , Vancaester E , et al. Asgard archaea illuminate the origin of eukaryotic cellular complexity. Nature. 2017;541(7637):353–358. 28077874 10.1038/nature21031

[56] Fukunaga R , Yokoyama S . Crystal structure of leucyl‐tRNA synthetase from the archaeon pyrococcus horikoshii reveals a novel editing domain orientation. J Mol Biol. 2005;346(1):57–71. 15663927 10.1016/j.jmb.2004.11.060

[57] Ribas de Pouplana L . The evolution of aminoacyl‐tRNA synthetases: from dawn to luca. Enzymes. 2020;48:11–37. 33837701 10.1016/bs.enz.2020.08.001

[58] Carter CW Jr , Wills PR . The roots of genetic coding in aminoacyl‐tRNA synthetase duality. Annu Rev Biochem. 2021;90:349–373. 33781075 10.1146/annurev-biochem-071620-021218

[59] Kondratyeva LG , Dyachkova MS , Galchenko AV . The origin of genetic code and translation in the framework of current concepts on the origin of life. Biochemistry (Moscow). 2022;87(2):150–169. 35508902 10.1134/S0006297922020079

[60] Rodin SN , Ohno S . Two types of aminoacyl‐tRNA synthetases could be originally encoded by complementary strands of the same nucleic acid. Orig Life Evol Biosph. 1995;25(6):565–589. 7494636 10.1007/BF01582025

[61] Carter CW , Li L , Weinreb V , Collier M , Gonzalez‐Rivera K , Jimenez‐Rodriguez M , et al. The Rodin‐Ohno hypothesis that two enzyme superfamilies descended from one ancestral gene: an unlikely scenario for the origins of translation that will not be dismissed. Biol Direct. 2014;9(1):1–23. 24927791 10.1186/1745-6150-9-11 PMC4082485

[62] Kauffman S , Lehman N . Mixed anhydrides at the intersection between peptide and RNA autocatalytic sets: evolution of biological coding. J R Soc Interface. 2023;13(3):20230009. 10.1098/rsfs.2023.0009 PMC10198252 37213924

[63] Hobson JJ , Li Z , Hu H , Carter CW Jr . A leucyl‐tRNA synthetase urzyme: authenticity of tRNA synthetase catalytic activities and promiscuous phosphorylation of leucyl‐5’ AMP. Int J Mol Sci. 2022;23(8):4229. 35457045 10.3390/ijms23084229 PMC9026127

[64] Li L , Weinreb V , Francklyn C , Carter CW . Histidyl‐tRNA synthetase urzymes: Class I and II aminoacyl tRNA synthetase urzymes have comparable catalytic activities for cognate amino acid activation. J Biol Chem. 2011;286(12):10387–10395. 21270472 10.1074/jbc.M110.198929 PMC3060492

[65] Tang GQ , Elder JJ , Douglas J , Carter CW Jr . Domain acquisition by class i aminoacyl‐tRNA synthetase urzymes coordinated the catalytic functions of hvgh and kmsks motifs. Nucleic Acids Res. 2023;51(15):8070–8084. 37470821 10.1093/nar/gkad590 PMC10450160

[66] Patra SK , Douglas J , Wills PR , Bouckeart R , Betts L , Qing TG , et al. Genomic database furnishes a spontaneous example of a functional class II glycyl‐tRNA synthetase urzyme. bioRxiv. 2024;2024–01. 10.1093/nar/gkae992 PMC11602164 39494520

[67] Onodera K , Suganuma N , Takano H , Sugita Y , Shoji T , Minobe A , et al. Amino acid activation analysis of primitive aminoacyl‐tRNA synthetases encoded by both strands of a single gene using the malachite green assay. Biosystems. 2021;208:104481. 34245865 10.1016/j.biosystems.2021.104481

[68] Tang T , Hu H , Douglas J , Carter C Jr . Primordial aminoacyl‐tRNA synthetases preferred minihelices to full‐length tRNA. Nucleic Acids Res. 2024;52:7096–7111. 38783009 10.1093/nar/gkae417 PMC11229368

[69] Francklyn C , Schimmel P . Aminoacylation of rna minihelices with alanine. Nature. 1989;337(6206):478–481. 2915692 10.1038/337478a0

[70] Schimmel P , Ribas de Pouplana L . Transfer RNA: from minihelix to genetic code. Cell. 1995;81(7):983–986. 7600584 10.1016/s0092-8674(05)80002-9

[71] Nordin BE , Schimmel P . RNA determinants for translational editing: mischarging a minihelix substrate by a tRNA synthetase. J Biol Chem. 1999;274(11):6835–6838. 10066735 10.1074/jbc.274.11.6835

[72] Koonin EV , Novozhilov AS . Origin and evolution of the genetic code: the universal enigma. IUBMB Life. 2009;61(2):99–111. 19117371 10.1002/iub.146 PMC3293468

[73] Wong JT‐F , Ng S‐K , Mat W‐K , Hu T , Xue H . Coevolution theory of the genetic code at age forty: pathway to translation and synthetic life. Life. 2016;6(1):12. 26999216 10.3390/life6010012 PMC4810243

[74] Illangasekare M , Sanchez G , Nickles T , Yarus M . Aminoacyl‐rna synthesis catalyzed by an rna. Science. 1995;267(5198):643–647. 7530860 10.1126/science.7530860

[75] Lohse PA , Szostak JW . Ribozyme‐catalysed amino‐acid transfer reactions. Nature. 1996;381(6581):442–444. 8632803 10.1038/381442a0

[76] Suga H , Hayashi G , Terasaka N . The rna origin of transfer rna aminoacylation and beyond. Philos Trans R Soc B: Biol Sci. 2011;366(1580):2959–2964. 10.1098/rstb.2011.0137 PMC3158918 21930588

[77] Jumper J , Evans R , Pritzel A , Green T , Figurnov M , Ronneberger O , et al. Highly accurate protein structure prediction with AlphaFold. Nature. 2021;596(7873):583–589. 34265844 10.1038/s41586-021-03819-2 PMC8371605

[78] Yin R , Feng BY , Varshney A , Pierce BG . Benchmarking alphafold for protein complex modeling reveals accuracy determinants. Protein Sci. 2022;31(8):e4379. 35900023 10.1002/pro.4379 PMC9278006

[79] Terwilliger TC , Liebschner D , Croll TI , Williams CJ , McCoy AJ , Poon BK , et al. AlphaFold predictions are valuable hypotheses and accelerate but do not replace experimental structure determination. Nat Methods. 2024;21(1):110–116. 38036854 10.1038/s41592-023-02087-4 PMC10776388

[80] Newberry KJ , Hou Y‐M , Perona JJ . Structural origins of amino acid selection without editing by cysteinyl‐tRNA synthetase. EMBO J. 2002;21(11):2778–2787. 12032090 10.1093/emboj/21.11.2778 PMC126036

[81] Chen B , Luo S , Zhang S , Ju Y , Gu Q , Xu J , et al. Inhibitory mechanism of reveromycin a at the tRNA binding site of a class i synthetase. Nat Commun. 2021;12(1):1616. 33712620 10.1038/s41467-021-21902-0 PMC7955072

[82] Rock FL , Mao W , Yaremchuk A , Tukalo M , Crépin T , Zhou H , et al. An antifungal agent inhibits an aminoacyl‐tRNA synthetase by trapping tRNA in the editing site. Science. 2007;316(5832):1759–1761. 17588934 10.1126/science.1142189

[83] Crepin T , Schmitt E , Mechulam Y , Sampson PB , Vaughan MD , Honek JF , et al. Use of analogues of methionine and methionyl adenylate to sample conformational changes during catalysis in *Escherichia coli* methionyl‐tRNA synthetase. J Mol Biol. 2003;332(1):59–72. 12946347 10.1016/s0022-2836(03)00917-3

[84] Fukai S , Nureki O , Sekine SI , Shimada A , Tao J , Vassylyev DG , et al. Structural basis for double‐sieve discrimination of l‐valine from l‐isoleucine and l‐threonine by the complex of tRNAVal and valyl‐tRNA synthetase. Cell. 2000;103(5):793–803. 11114335 10.1016/s0092-8674(00)00182-3

[85] Rath VL , Silvian LF , Beijer B , Sproat BS , Steitz TA . How glutaminyl‐tRNA synthetase selects glutamine. Structure. 1998;6(4):439–449. 9562563 10.1016/s0969-2126(98)00046-x

[86] Sekine S‐i , Nureki O , Dubois DY , Bernier S , Chênevert R , Lapointe J , et al. ATP binding by glutamyl‐tRNA synthetase is switched to the productive mode by tRNA binding. EMBO J. 2003;22(3):676–688. 12554668 10.1093/emboj/cdg053 PMC140737

[87] Schulze JO , Masoumi A , Nickel D , Jahn M , Jahn D , Schubert WD , et al. Crystal structure of a non‐discriminating glutamyl‐tRNA synthetase. J Mol Biol. 2006;361(5):888–897. 16876193 10.1016/j.jmb.2006.06.054

[88] Chung S , Kang MS , Alimbetov DS , Mun GI , Yunn NO , Kim Y , et al. Regulation of brca1 stability through the tandem ubx domains of isoleucyl‐tRNA synthetase 1. Nat Commun. 2022;13(1):6732. 36347866 10.1038/s41467-022-34612-y PMC9643514

[89] Nureki O , O'Donoghue P , Watanabe N , Ohmori A , Oshikane H , Araiso Y , et al. Structure of an archaeal non‐discriminating glutamyl‐tRNA synthetase: a missing link in the evolution of gln‐tRNAGln formation. Nucleic Acids Res. 2010;38(20):7286–7297. 20601684 10.1093/nar/gkq605 PMC2978374

[90] Retailleau P , Yin Y , Hu M , Roach J , Bricogne G , Vonrhein C , et al. High‐resolution experimental phases for tryptophanyl‐tRNA synthetase (TrpRS) complexed with tryptophanyl‐5’ AMP. Acta Crystallogr D Biol Crystallogr. 2001;57(11):1595–1608. 11679724 10.1107/s090744490101215x

[91] Abergel C , Rudinger‐Thirion J , Giegé R , Claverie J‐M . Virus‐encoded aminoacyl‐tRNA synthetases: structural and functional characterization of mimivirus tyrrs and metrs. J Virol. 2007;81(22):12406–12417. 17855524 10.1128/JVI.01107-07 PMC2169003

[92] Cavarelli J , Delagoutte B , Eriani G , Gangloff J , Moras D . L‐arginine recognition by yeast arginyl‐tRNA synthetase. EMBO J. 1998;17(18):5438–5448. 9736621 10.1093/emboj/17.18.5438 PMC1170869

[93] Logan D , Mazauric M , Kern D , Moras D . Crystal structure of glycyl‐tRNA synthetase from *Thermus thermophilus* . EMBO J. 1995;14(17):4156–4167. 7556056 10.1002/j.1460-2075.1995.tb00089.x PMC394498

[94] Cader MZ , Ren J , James PA , Bird LE , Talbot K , Stammers DK . Crystal structure of human wildtype and s581l‐mutant glycyl‐tRNA synthetase, an enzyme underlying distal spinal muscular atrophy. FEBS Lett. 2007;581(16):2959–2964. 17544401 10.1016/j.febslet.2007.05.046

[95] Kamtekar S , Kennedy WD , Wang J , Stathopoulos C , Söll D , Steitz TA . The structural basis of cysteine aminoacylation of tRNAPro by prolyl‐tRNA synthetases. Proc Natl Acad Sci. 2003;100(4):1673–1678. 12578991 10.1073/pnas.0437911100 PMC149891

[96] Chimnaronk S , Gravers Jeppesen M , Suzuki T , Nyborg J , Watanabe K . Dual‐mode recognition of noncanonical tRNAsSer by seryl‐tRNA synthetase in mammalian mitochondria. EMBO J. 2005;24(19):3369–3379. 16163389 10.1038/sj.emboj.7600811 PMC1276171

[97] Bilokapic S , Maier T , Ahel D , Gruic‐Sovulj I , Söll D , Weygand‐Durasevic I , et al. Structure of the unusual seryl‐tRNA synthetase reveals a distinct zinc‐dependent mode of substrate recognition. EMBO J. 2006;25(11):2498–2509. 16675947 10.1038/sj.emboj.7601129 PMC1478180

[98] Torres‐Larios A , Sankaranarayanan R , Rees B , Dock‐Bregeon A‐C , Moras D . Conformational movements and cooperativity upon amino acid, atp and tRNA binding in threonyl‐tRNA synthetase. J Mol Biol. 2003;331(1):201–211. 12875846 10.1016/s0022-2836(03)00719-8

[99] Iwasaki W , Sekine S‐i , Kuroishi C , Kuramitsu S , Shirouzu M , Yokoyama S . Structural basis of the water‐assisted asparagine recognition by asparaginyl‐tRNA synthetase. J Mol Biol. 2006;360(2):329–342. 16753178 10.1016/j.jmb.2006.04.068

[100] Eiler S , Dock‐Bregeon A‐C , Moulinier L , Thierry J‐C , Moras D . Synthesis of aspartyl‐tRNAAsp in *Escherichia coli*—a snapshot of the second step. EMBO J. 1999;18(22):6532–6541. 10562565 10.1093/emboj/18.22.6532 PMC1171716

[101] Sauter C , Lorber B , Cavarelli J , Moras D , Giegé R . The free yeast aspartyl‐tRNA synthetase differs from the tRNAAsp‐complexed enzyme by structural changes in the catalytic site, hinge region, and anticodon‐binding domain. J Mol Biol. 2000;299(5):1313–1324. 10873455 10.1006/jmbi.2000.3791

[102] Schmitt E , Moulinier L , Fujiwara S , Imanaka T , Thierry J‐C , Moras D . Crystal structure of aspartyl‐tRNA synthetase from Pyrococcus kodakaraensis KOD: archaeon specificity and catalytic mechanism of adenylate formation. EMBO J. 1998;17(17):5227–5237. 9724658 10.1093/emboj/17.17.5227 PMC1170850

[103] Sakurama H , Takita T , Mikami B , Itoh T , Yasukawa K , Inouye K . Two crystal structures of lysyl‐tRNA synthetase from bacillus stearothermophilus in complex with lysyladenylate‐like compounds: insights into the irreversible formation of the enzyme‐bound adenylate of l‐lysine hydroxamate. J Biochem. 2009;145(5):555–563. 19174549 10.1093/jb/mvp014

[104] Finarov I , Moor N , Kessler N , Klipcan L , Safro MG . Structure of human cytosolic phenylalanyl‐tRNA synthetase: evidence for kingdom‐specific design of the active sites and tRNA binding patterns. Structure. 2010;18(3):343–353. 20223217 10.1016/j.str.2010.01.002

[105] Mermershtain I , Finarov I , Klipcan L , Kessler N , Rozenberg H , Safro MG . Idiosyncrasy and identity in the prokaryotic phe‐system: crystal structure of *E. coli* phenylalanyl‐tRNA synthetase complexed with phenylalanine and AMP. Protein Sci. 2011;20(1):160–167. 21082706 10.1002/pro.549 PMC3047072

[106] Klipcan L , Levin I , Kessler N , Moor N , Finarov I , Safro M . The tRNA‐induced conformational activation of human mitochondrial phenylalanyl‐tRNA synthetase. Structure. 2008;16(7):1095–1104. 18611382 10.1016/j.str.2008.03.020

[107] Arnez J , Harris D , Mitschler A , Rees B , Francklyn C , Moras D . Crystal structure of histidyl‐tRNA synthetase from *Escherichia coli* complexed with histidyl‐adenylate. EMBO J. 1995;14(17):4143–4155. 7556055 10.1002/j.1460-2075.1995.tb00088.x PMC394497

[108] Kamtekar S , Hohn MJ , Park HS , Schnitzbauer M , Sauerwald A , Söll D , et al. Toward understanding phosphoseryl‐tRNACys formation: the crystal structure of methanococcus maripaludis phosphoseryl‐tRNA synthetase. Proc Natl Acad Sci. 2007;104(8):2620–2625. 17301225 10.1073/pnas.0611504104 PMC1815232

[109] Kavran JM , Gundllapalli S , O'Donoghue P , Englert M , Söll D , Steitz TA . Structure of pyrrolysyl‐tRNA synthetase, an archaeal enzyme for genetic code innovation. Proc Natl Acad Sci. 2007;104(27):11268–11273. 17592110 10.1073/pnas.0704769104 PMC2040888

[110] Mirdita M , Schütze K , Moriwaki Y , Heo L , Ovchinnikov S , Steinegger M . ColabFold: making protein folding accessible to all. Nat Methods. 2022;19(6):679–682. 35637307 10.1038/s41592-022-01488-1 PMC9184281

[111] Kabsch W , Sander C . Dictionary of protein secondary structure: pattern recognition of hydrogen‐bonded and geometrical features. Biopolymers. 1983;22(12):2577–2637. 6667333 10.1002/bip.360221211

[112] Joosten RP , te Beek TAH , Krieger E , Hekkelman ML , Hooft RWW , Schneider R , et al. A series of pdb related databases for everyday needs. Nucleic Acids Res. 2010;39(Suppl 1):D411–D419. 21071423 10.1093/nar/gkq1105 PMC3013697

[113] Wang S , Ma J , Peng J , Xu J . Protein structure alignment beyond spatial proximity. Sci Rep. 2013;3(1):1–7. 10.1038/srep01448 PMC3596798 23486213

[114] Wang S , Peng J , Xu J . Alignment of distantly related protein structures: algorithm, bound and implications to homology modeling. Bioinformatics. 2011;27(18):2537–2545. 21791532 10.1093/bioinformatics/btr432 PMC3167051

[115] Thompson JD , Gibson TJ , Higgins DG . Multiple sequence alignment using ClustalW and ClustalX. Curr Protoc Bioinformatics. 2003;1:2–3. 10.1002/0471250953.bi0203s00 18792934

[116] Lin BY , Chan PP , Lowe TM . TRNAviz: explore and visualize tRNA sequence features. Nucleic Acids Res. 2019;47(W1):W542–W547. 31127306 10.1093/nar/gkz438 PMC6602477

[117] Bouckaert R , Vaughan TG , Barido‐Sottani J , Duchêne S , Fourment M , Gavryushkina A , et al. BEAST 2.5: an advanced software platform for Bayesian evolutionary analysis. PLoS Comput Biol. 2019;15(4):e1006650. 30958812 10.1371/journal.pcbi.1006650 PMC6472827

[118] Douglas J , Zhang R , Bouckaert R . Adaptive dating and fast proposals: revisiting the phylogenetic relaxed clock model. PLoS Comput Biol. 2021;17(2):e1008322. 33529184 10.1371/journal.pcbi.1008322 PMC7880504

[119] Bouckaert RR . OBAMA: OBAMA for Bayesian amino‐acid model averaging. PeerJ. 2020;8:e9460. 32832259 10.7717/peerj.9460 PMC7413081

[120] Bouckaert RR . An efficient coalescent epoch model for Bayesian phylogenetic inference. Syst Biol. 2022;71(6):1549–1560. 10.1093/sysbio/syac015 35212733 PMC9773037

[121] Berling L , Klawitter J , Bouckaert R , Xie D , Gavryushkin A , Drummond A . Accurate Bayesian phylogenetic point estimation using a tree distribution parameterized by clade probabilities. bioRxiv. 2024;2024–02. 10.1371/journal.pcbi.1012789 PMC11835378 39937844

[122] Rambaut A , Drummond AJ , Xie D , Baele G , Suchard MA . Posterior summarization in Bayesian phylogenetics using tracer 1.7. Syst Biol. 2018;67(5):901–904. 29718447 10.1093/sysbio/syy032 PMC6101584

[123] Whelan S , Goldman N . A general empirical model of protein evolution derived from multiple protein families using a maximum‐likelihood approach. Mol Biol Evol. 2001;18(5):691–699. 11319253 10.1093/oxfordjournals.molbev.a003851

[124] Le SQ , Gascuel O . An improved general amino acid replacement matrix. Mol Biol Evol. 2008;25(7):1307–1320. 18367465 10.1093/molbev/msn067

[125] Kobayashi T , Takimura T , Sekine R , Vincent K , Kamata K , Sakamoto K , et al. Structural snapshots of the kmsks loop rearrangement for amino acid activation by bacterial tyrosyl‐tRNA synthetase. J Mol Biol. 2005;346(1):105–117. 15663931 10.1016/j.jmb.2004.11.034

[126] Giegé R , Eriani G . The tRNA identity landscape for aminoacylation and beyond. Nucleic Acids Res. 2023;51(4):1528–1570. 36744444 10.1093/nar/gkad007 PMC9976931

[127] Eriani G , Delarue M , Poch O , Gangloff J , Moras D . Partition of tRNA synthetases into two classes based on mutually exclusive sets of sequence motifs. Nature. 1990;347(6289):203–206. 2203971 10.1038/347203a0

[128] Szymanski M , Deniziak MA , Barciszewski J . Aminoacyl‐tRNA synthetases database. Nucleic Acids Res. 2001;29(1):288–290. 11125115 10.1093/nar/29.1.288 PMC29805

[129] Chaliotis A , Vlastaridis P , Mossialos D , Ibba M , Becker HD , Stathopoulos C , et al. The complex evolutionary history of aminoacyl‐tRNA synthetases. Nucleic Acids Res. 2017;45(3):1059–1068. 28180287 10.1093/nar/gkw1182 PMC5388404

[130] Osawa S , Jukes T , Watanabe K , Muto A . Recent evidence for evolution of the genetic code. Microbiol Rev. 1992;56(1):229–264. 1579111 10.1128/mr.56.1.229-264.1992 PMC372862

[131] Jukes T , Osawa S . Evolutionary changes in the genetic code. Comp Biochem Physiol. 1993;106(3):489–494. 10.1016/0305-0491(93)90122-l 8281749

[132] Placzek S , Schomburg I , Chang A , Jeske L , Ulbrich M , Tillack J , et al. BRENDA in 2017: new perspectives and new tools in brenda. Nucleic Acids Res. 2016;45(D1):D380–D388. 27924025 10.1093/nar/gkw952 PMC5210646

[133] Alvarez‐Carreño C , Arciniega M , Ribas de Pouplana L , Petrov AS , Hernández‐González A , Dimas‐Torres JU , et al. Common evolutionary origins of the bacterial glycyl tRNA synthetase and alanyl tRNA synthetase. Protein Sci. 2024;33(3):e4844. 10.1002/pro.4844 PMC10895455 38009704

[134] Abramson J , Adler J , Dunger J , Evans R , Green T , Pritzel A , et al. Accurate structure prediction of biomolecular interactions with AlphaFold 3. Nature. 2024;630(8016):493–500. 38718835 10.1038/s41586-024-07487-w PMC11168924

